# Macrophage Migration Inhibitory Factor (MIF) Expression Increases during Myocardial Infarction and Supports Pro-Inflammatory Signaling in Cardiac Fibroblasts

**DOI:** 10.3390/biom9020038

**Published:** 2019-01-23

**Authors:** Svenja Voss, Saskia Krüger, Katharina Scherschel, Svenja Warnke, Michael Schwarzl, Benedikt Schrage, Evaldas Girdauskas, Christian Meyer, Stefan Blankenberg, Dirk Westermann, Diana Lindner

**Affiliations:** 1 Clinic for General and Interventional Cardiology, University Heart Center Hamburg, University Hospital Hamburg-Eppendorf, 20246 Hamburg, Germany; Svenja.voss@yahoo.com (S.V.); s.krueger@uke.de (S.K.); s.warnke@uke.de (S.W.); m.schwarzl@uke.de (M.S.); b.schrage@uke.de (B.S.); s.blankenberg@uke.de (S.B.); d.westermann@uke.de (D.W.); 2 Partner Site Hamburg/Kiel/Lübeck, DZHK (German Center for Cardiovascular Research), 20246 Hamburg, Germany; k.scherschel@uke.de (K.S.); e.girdauskas@uke.de (E.G.); chr.meyer@uke.de (C.M.); 3 Clinic for Cardiology—Electrophysiology, University Heart Center Hamburg, University Hospital Hamburg-Eppendorf, 20246 Hamburg, Germany; 4 Clinic for Cardiovascular Surgery, University Heart Center Hamburg, University Hospital Hamburg-Eppendorf, 20246 Hamburg, Germany

**Keywords:** myocardial infarction, macrophage migration inhibitory factor, MIF-2, cardiac fibroblasts, cardiac inflammation

## Abstract

Macrophage migration inhibitory factor (MIF) is a pleiotropic cytokine known to play a major role in inflammatory diseases such as myocardial infarction (MI), where its expression increases. Cardio-protective functions of MIF during ischemia have been reported. Recently, the structurally related MIF-2 was identified and similar effects are assumed. We wanted to further investigate the role of MIF and MIF-2 on inflammatory processes during MI. Therefore, we subjected mice to experimentally induced MI by coronary occlusion for one and five days. During the acute phase of MI, the gene expression of *Mif* was upregulated in the infarct zone, whereas *Mif-2* was downregulated, suggesting a minor role of MIF-2. Simulating ischemic conditions or mechanical stress in vitro, we demonstrated that *Mif* expression was induced in resident cardiac cells. To investigate possible auto-/paracrine effects, cardiomyocytes and cardiac fibroblasts were individually treated with recombinant murine MIF, which in turn induced *Mif* expression and the expression of pro-inflammatory genes in cardiac fibroblasts. Cardiomyocytes did not respond to recombinant MIF with pro-inflammatory gene expression. While MIF stimulation alone did not change the expression of pro-fibrotic genes in cardiac fibroblasts, ischemia reduced their expression. Mimicking the increased MIF levels during MI, we exposed cardiac fibroblasts to simulated ischemia in the presence of MIF, which led to further reduced expression of pro-fibrotic genes. The presented data show that MIF was expressed by resident cardiac cells during MI. In vitro, *Mif* expression was induced by different external stimuli in cardiomyocytes and cardiac fibroblasts. Addition of recombinant MIF protein increased the expression of pro-inflammatory genes in cardiac fibroblasts including *Mif* expression itself. Thereby, cardiac fibroblasts may amplify *Mif* expression during ischemia promoting cardiomyocyte survival.

## 1. Introduction

Myocardial infarction (MI) remains one of the leading causes of death in Western society [1,2]. The acute interruption of blood flow to cardiac tissue leads to a demise of cardiomyocytes within the ischemic area [1,3]. Necrotic cardiomyocytes release a wide range of inflammatory molecules, which rapidly activate the innate immune response. This, further triggers the infiltration of the infarcted heart by leukocytes, leading to local inflammatory processes [3,4,5]. Recruited leukocytes remove dead cells and cellular debris via phagocytosis. This cardiac inflammatory response is thereby essential for cardiac repair and promotes scar formation by deposition of extracellular matrix proteins. However, aggravated cardiac inflammation can induce adverse tissue remodeling, which also impairs contractile function and can finally lead to the development of heart failure [3,6,7,8,9].

The macrophage migration inhibitory factor (MIF) is a 114 amino acid protein, structured as a trimer of identical subunits [10,11]. Since MIF is known as a pleiotropic inflammatory cytokine, it is involved in several inflammatory diseases such as MI, atherosclerosis, rheumatoid arthritis and sepsis [12,13,14,15]. First discovered as a T-cell-derived cytokine that inhibits the migration of macrophages [16,17,18], based on today’s knowledge, MIF is expressed by immune and non-immune cells, including cardiomyocytes, and mediates the recruitment of mononuclear cells [18,19,20,21,22]. Its functions are mediated via the CD74/CD44 complex, but it is also described as a non-cognate ligand of the CXC chemokine receptors, CXCR2 and CXCR4 [23,24,25]. Recently, a second member of the MIF family was identified. Despite only 27% identity of the amino acid sequence, MIF and D-dopachrome tautomerase (D-DT, MIF-2) share a highly conserved structure. Similarly, it acts as homotrimer through the same cell surface receptors and is therefore designated as MIF-2 [26,27].

Elevated plasma levels of MIF are detectable in patients after acute MI for up to two weeks [18,28,29] and can serve as a biomarker to predict infarct size. High MIF plasma levels on hospital admission are correlated with larger infarct size [30]. During a short ischemic/reperfusion setting, *Mif*^-/-^ deficient mice showed increased infarct size, suggesting a cardio-protective function of MIF [31]. Given the contribution to inflammation during prolonged ischemia/reperfusion, MIF deficiency protects the heart and reduces infarct size [32]. However, in cultured cardiomyocytes, MIF expression is induced by hypoxia and redox stress which may further indicate a cardio-protective function [18,20,33]. Here, we investigated *Mif* and *Mif-2* expression after myocardial infarction induced by coronary occlusion and identified cardiac fibroblasts as a more prominent cellular source of *Mif* within the infarct zone. In in vitro experiments, we applied various external stimuli to cardiomyocytes and cardiac fibroblasts to examine *Mif* expression and further its function regarding pro-inflammatory and pro-fibrotic signaling pathways.

## 2. Materials and Methods

### 2.1. Animal Model

Male C57BL/6J (B6) mice were used at the age of 8 to 10 weeks for the presented animal mouse model. Mice were anesthetized under artificial ventilation using isoflurane and buprenorphine was given as analgesic therapy. Access to the heart was gained via the third left intercostal space. MI was induced by permanent ligation of the left anterior descending artery (LAD) as described previously [34]. Sham mice underwent the same procedure except for coronary ligation. Mice were sacrificed after one day to analyze the acute phase (6 sham, 5 MI) or after five days to analyze the subacute phase (8 sham, 11 MI). Hearts were removed and remote zone and border zone were separated from infarcted tissue. All tissue samples were snap frozen in liquid nitrogen and stored at −80 °C for further analyses.

All investigations were approved by the local bioethics committee of Hamburg, Germany (G15/060) and conform to the Guide for the Care and Use of Laboratory Animals published by the US NIH (NIH Publication number 85-23, revised 1996).

### 2.2. Cell Culture

The hearts of male wildtype C57BL/6J mice (10–12 weeks old) were used to obtain primary murine cardiac fibroblasts from the left ventricle (LV) as described previously [6]. The LV tissue was cut into pieces and digested in 0.1 mg/mL liberase (Roche, Grenzach-Wyhlen, Germany) under gentle shaking at 37 °C for 10 min. The cell-containing supernatant was collected and immediately stored on ice. The remaining tissue was further digested by addition of liberase for 10 min. This procedure was repeated six times. In order to remove cell aggregates, the cell-containing supernatant was subsequently filtered through a cell strainer to obtain a single-cell solution. To remove the digesting buffer, cells were pelletized and resuspended in complete growth medium (DMEM containing 20% fetal calf serum (FCS), 100 U/mL penicillin and 100 µg/mL streptomycin (Sigma-Aldrich, St. Louis, MO, USA) and cultured in a humidified atmosphere at 37 °C, 5% carbon dioxide and 95% air. For sub-culturing, cells were detached utilizing trypsin/EDTA (ethylenediaminetetraacetic acid) solution. To verify that we obtained cardiac fibroblasts, specific antibodies were used for positive and negative staining: cells were positive for collagen-I and negative for the myocyte marker desmin as well as the endothelial marker CD31, as described previously [6].

The HL-1 cells, a well-established murine cardiomyocyte cell line, were cultured as recommended in Claycomb medium (Sigma-Aldrich) supplemented with 10% FCS, 100 U/mL penicillin, 100 µg/mL streptomycin, 2 mM L-glutamine and 0.1 µM norepinephrine (Sigma-Aldrich) [35].

Murine splenocytes were isolated from freshly removed spleen. To obtain a single-cell solution, the spleen was mashed through a cell strainer. The cell strainer was rinsed using phosphate-buffered saline (PBS) buffer, which was removed after centrifugation. The obtained cell pellet was resuspended in 3 mL PBS and layered onto 3 mL of Histopaque-1077 (Sigma-Aldrich) in a 15 mL conical tube and centrifuged according to the manufacturer’s protocol. Supernatant was removed after centrifugation and the remaining cell pellet, containing the splenocytes, was collected in RPMI medium supplemented with 10% FCS, 100 U/mL penicillin and 100 µg/mL streptomycin (Sigma-Aldrich). Experiments with these cells were performed immediately.

### 2.3. Simulated Ischemia, Mechanical Stretch and Cytokine Stimulation in Cell Culture Experiments

Cardiac fibroblasts or cardiomyocytes were seeded in 12-well plates and grown to confluence before starving overnight in Dulbecco’s modified eagle’s medium (DMEM) containing 0.5% FCS, 100 U/mL penicillin and 100 µg/mL streptomycin (Sigma-Aldrich).

To induce ischemic conditions, cells received starving medium containing 4.5 g/L L-glucose instead of D-glucose and were exposed to a gas mixture of 94% nitrogen, 5% carbon dioxide and 1% oxygen. Therefore, cells were placed into a modular incubator chamber (MIC-101, Billups-Rothenberg, Inc., San Diego, CA, USA), which was flushed with nitrogen and carbon dioxide using a flow rate ratio of 20 to 1, respectively until the final oxygen concentration of 1% was reached, detected by an oxygen sensor (Greisinger, Würzburg, Germany).

Mechanical stretch was applied to cardiomyocytes or cardiac fibroblasts using the Flexcell^®^ FX-4000™ Tension System (Dunn Labortechnik, Asbach, Germany). Therefore, cells were seeded in flexible-bottomed 6-well culture plates (Bioflex plates) coated with collagen-I (Flexcell Int. Corp., Burlington, NC, USA). When cells reached confluence, they were exposed to mechanical stretch regulated by vacuum pressure to deform the cultured cells. Mechanical tension was applied resulting in 10% elongation at a frequency of 1 Hz for 6 h. Non-stretched control cells were seeded in the same flexercell plates without exposing them to mechanical stretch.

Stimulation was performed using a final concentration of 50 ng/mL recombinant murine MIF (R&D Systems, Minneapolis, MN, USA) diluted in starving medium. Cardiomyocytes or cardiac fibroblasts were incubated with or without MIF protein for 24 h prior to RNA isolation. Furthermore, some cell culture samples were exposed to ischemic conditions and at the same time stimulated with MIF protein. In this case, MIF was diluted in starving medium containing 4.5 g/L L-glucose instead of D-glucose.

To induce pro-inflammatory or pro-fibrotic signaling, cardiac fibroblasts were starved overnight prior to stimulation using recombinant 10 ng/mL Tumor Necrosis Factor (TNF)-α (Peprotech, Hamburg, Germany) or 5 ng/mL transforming growth factor-beta (TGF)-β (Peprotech), respectively. Recombinant proteins were diluted in starving medium, added to cardiac fibroblasts and incubated for 6 h prior to RNA isolation. Non-stimulated control cells were treated equally without addition of the recombinant proteins.

To examine activated leukocytes, freshly isolated splenocytes were used and activated with cell culture supernatant derived from ischemic cardiac fibroblasts for 24 h. Non-stimulated control cells were incubated in starving medium.

### 2.4. Histology and Immunofluorescence

Immunohistological staining of infarcted hearts was performed as previously described [36]. Briefly, after routine processing for paraffin embedding, the hearts were cut in 4 µm sections. Antigen retrieval was performed in a pressure cooker with citrate buffer pH 6. After incubation in 0.25% sudan black B (C.I. 26150, Roth) in 70% ethanol and subsequent permeabilization in 0.2% triton X-100, sections were blocked in 3% bovine serum albumin/tris-buffered saline (BSA/TBS) at room temperature for 1 h. Primary antibodies (Table 1) were incubated overnight in 1% BSA/TBS. After washing, incubation with the appropriate Alexa-labelled secondary antibodies and Alexa 633-labelled wheat germ agglutinin (WGA) (W21404, Life Technologies, Carlsbad, CA, USA) was performed. Sections were imaged with a Leica TCS SP8 confocal microscope and maximum projection images were created using the Leica LAS X SP8 software (Leica, Wetzlar, Germany).

### 2.5. RNA Isolation

Total RNA from frozen cardiac tissue samples was isolated using QIAzol lysis reagent. Disruption of the tissue was achieved using pellet pestles followed by vigorous shaking for 10 min. To obtain the RNA, chloroform was added. After centrifugation, the upper RNA-containing phase was collected and ethanol was added, followed by further purification using the miRNeasy mini kit (Qiagen, Hilden, Germany). To obtain total RNA from cells, RNeasy Mini Kit (Qiagen) was used according to the manufacturer’s protocol. To avoid genomic DNA contamination within the isolated RNA from tissue or cells, DNase-I (Qiagen) was applied directly on the column during the purification protocol. The concentration of the resulting RNA was determined by measuring the absorbance at 260 nm using the Nanodrop 2000c spectrophotometer (Thermo Fisher Scientific, Waltham, MA, USA). RNA was stored at −80 °C for further processing.

### 2.6. Reverse Transcription and Gene Expression Analysis

Reverse transcription of RNA was carried out using the High-capacity cDNA kit (Life technologies). An amount of 250 ng total RNA from cell culture experiments or 1 µg from tissue samples was reversely transcribed into cDNA for 2 h at 37 °C, followed by an inactivation step of 5 min at 85 °C. The resulting cDNA was further diluted to a final working concentration of 1.25 ng/µL for cell culture samples and 10 ng/µL for tissue samples.

Real-time PCR was performed to assess gene expression for target genes using 5 µL gene expression master mix (Life technologies) and 0.5 µL gene expression assay (Table 2) purchased from Life technologies. A gene expression assay includes forward and reverse primers as well as the fluorescently labelled probe. A volume of 1 µL of cDNA was used as template in a final volume of 10 µL. Each sample was analyzed in duplicates. Furthermore, the gene expression of *Cdkn1b* was determined as endogenous control to normalize the data using the formula 2^−∆∆Ct^ and plotted as x-fold to *Cdkn1b* as absolute gene expression. The relative gene expression data were additionally normalized using the formula 2^−∆∆Ct^ and plotted as x-fold to the untreated control as described previously [37]. Real-time PCR was performed on a 7900 TaqMan system using the software SDS v2.4 (Applied Biosystems, Foster City, CA, USA).

### 2.7. Statistics

Data are presented as box plots. All data were analyzed using GraphPad Prism 6 software (GraphPad Software, La Jolla, CA, USA). Statistical comparison of two groups was performed using the Mann-Whitney U test with *p* values < 0.05 considered statistically significant. More than two groups were compared using the Kruskal–Wallis test followed by Dunn’s post-test with *p* values < 0.05 considered statistically significant. Pearson’s correlation coefficients were computed by GraphPad Prism 6.

## 3. Results

### 3.1. Expression of Macrophage Migration Inhibitory Factor is Upregulated in Mice After Myocardial Infarction

**Figure 1:**
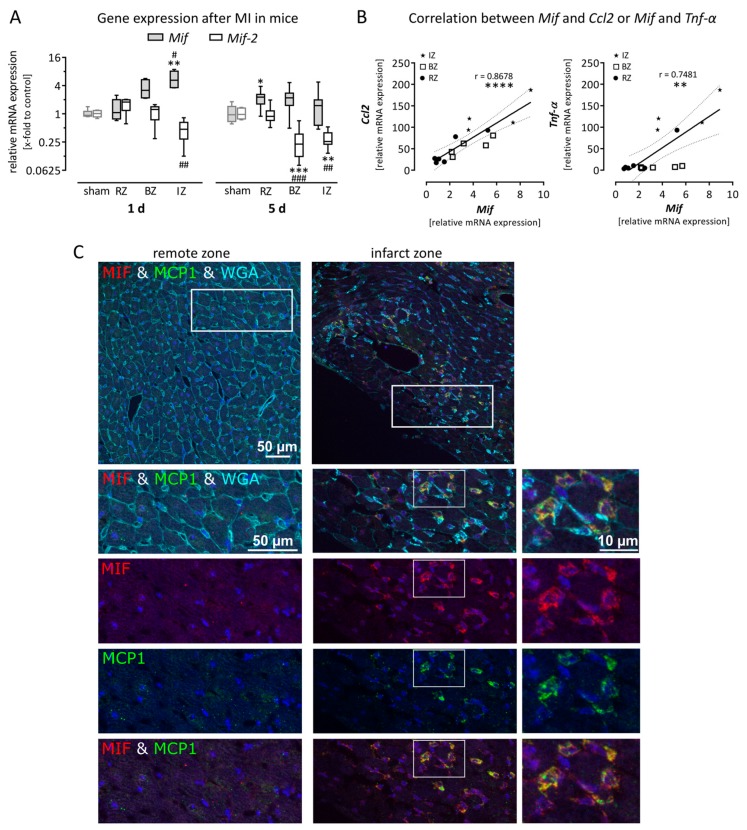
*Mif* and *Mif-2* expression is oppositely regulated in mice after Myocardial infarction (MI). (**A**) MI was induced in mice by permanent coronary ligation. Gene expression of *Mif* and *Mif-2* was quantified in left ventricular (LV) tissue samples collected from the remote zone (RZ), border zone (BZ) and infarct zone (IZ). Gene expression in the IZ was oppositely regulated for the two genes. While *Mif* was significantly increased during the acute phase (1 day) after MI, *Mif-2* gene expression was significantly decreased. However, 5 days after MI, *Mif* gene expression returned to basal levels, whereas *Mif-2* expression remained decreased. (**B**) Gene expression of *Ccl2* and *Tnf-α* was quantified in LV tissue samples collected 1 day after MI. Gene expression of *Mif* is plotted on the x-axis while *Ccl2* and *Tnf-α* are plotted on the y-axis. Data collected from the IZ are shown as an asterisk, from the BZ as open squares and from the RZ as filled circles. *Mif* expression highly correlated to the expression of *Ccl2* and *Tnf-α*. (**C**) Immunofluorescent staining of the RZ and the IZ of a cross section 1 day after MI is shown. The WGA staining was used to visualize the structural integrity of cardiac tissue and revealed a disarranged myocardial architecture within the IZ. Protein expression of MIF (red) and MCP-1 (green) are visible at higher magnifications. While no MIF expression and very low MCP-1 expression were determined in the RZ, both cytokines are highly expressed in the IZ. Furthermore, the overlay of the co-staining (yellow) revealed that both cytokines are co-localized in the same cells. Gene expression levels are shown as relative mRNA expression (x-fold to sham operated mice) using the formula 2^−ΔΔCt^ and presented in box plots (min to max). Gene expression data represent 6–8 sham operated mice and 5–11 mice with MI. Scale bar represents 50 µm or 10 µm. * significantly different compared with sham operated mice. * *p* < 0.05; ** *p* < 0.01; *** *p* < 0.001; **** *p* < 0.0001; # significantly different compared with remote zone, # *p* < 0.05; ## *p* < 0.01; ### *p* < 0.001.

Myocardial Infarction was induced in mice by permanent LAD ligation. Tissue samples were taken from the remote zone (RZ), the border zone (BZ) and the infarct zone (IZ) of the LV after the acute phase (1 day) and the subacute phase (5 days). The gene expression of *Mif* and *Mif-2* was quantified and plotted as relative mRNA expression compared with sham operated animals. Figure 1A shows that gene expression of *Mif* was increased in the BZ and IZ during the acute phase after MI, however significant upregulation was determined only in the IZ (5.8 ± 1.0-fold). In contrast, the gene expression of *Mif-2* was decreased in the IZ (0.5 ± 0.2-fold). The upregulation of *Mif* in the IZ returned to basal levels after 5 days (1.5 ± 0.4-fold), whereas *Mif-2* expression remained decreased 5 days after MI.

Additionally, gene expression of the pro-inflammatory markers *Ccl2* and *Tnf-α* was determined, representing the inflammation process during the acute phase after MI. Using linear regression, the gene expression of both genes was plotted against *Mif* expression 1 day after MI. As shown in Figure 1B, the gene expression of *Mif* highly correlates with the expression of *Ccl2* (r = 0.8678) and *Tnf-α* (r = 0.7481). To verify these mRNA data, immunofluorescence staining of cardiac tissue collected 1 day after MI was performed. As shown in Figure 1C, the myocardial architecture was clearly disorganized within the infarct zone, demonstrated by WGA staining. Furthermore, protein expression of MIF (red) and MCP-1 (green) was increased within the infarct zone compared with the remote zone during the acute phase (1 day) after MI. The overlay (yellow) after the co-staining revealed that both cytokines are expressed in the same cells.

### 3.2. Gene Expression of Mif Receptors Cxcr4 as Well as Cd74/Cd44 Was Increased after Myocardial Infarction as a Result of Recruited Leukocytes

**Figure 2:**
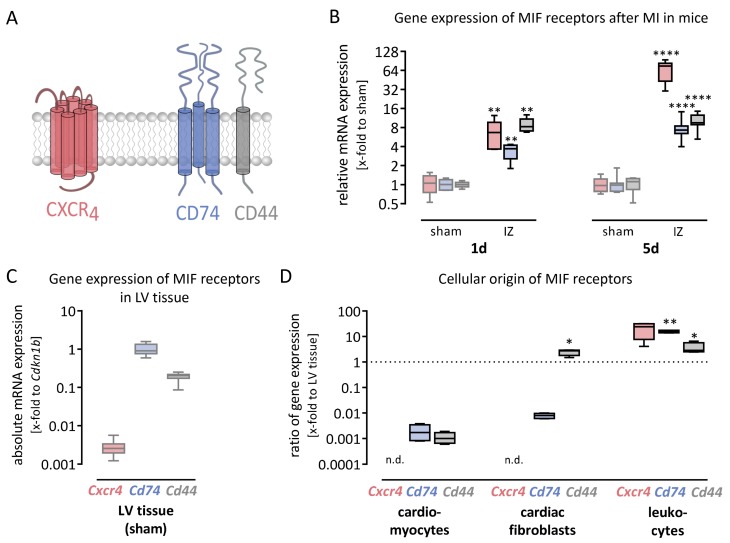
MIF receptors *Cxcr4* and *Cd74/Cd44* are expressed in cardiac tissue and upregulated after MI as a result of recruited leukocytes. (**A**) MIF and MIF-2 can bind to the G protein-coupled receptor CXCR4 (red) as well as the heterodimer CD74/CD44 (blue/grey) leading to different intracellular signaling. (**B**) The gene expression levels of all three transmembrane proteins were highly increased after myocardial infarction. Gene expression levels are shown as relative mRNA expression (x-fold to sham operated mice) using the formula 2^−ΔΔCt^ and presented in box plots (min to max). Gene expression data represent 6–8 sham operated mice and 5–11 mice with MI. (**C**) The gene expression levels of all three transmembrane proteins were determined in healthy LV tissue collected from mice after sham operation. While the expression of *Cxcr4* is very low, *Cd74* and *Cd44* forming the heterodimeric MIF receptor were well expressed in healthy LV tissue. Basal gene expression levels are shown as absolute mRNA expression (x-fold to the housekeeping gene *Cdkn1b*) using the formula 2^−ΔCt^ and presented in box plots (min to max). (**D**) The basal gene expression levels of *Cxcr4*, *Cd74* and *Cd44* were determined in cardiomyocytes, cardiac fibroblasts and leukocytes. To evaluate the cellular origin of the receptor expression in LV tissue, the ratio between the detected gene expression in the distinct cell type and the expression level in LV tissue was plotted. Those cells with a ratio lower than one contribute less to the receptor expression detected in the whole cardiac tissue. As depicted in red, the *Cxcr4* receptor is not expressed in both resident cardiac cell types, cardiomyocytes and cardiac fibroblasts, whereas leukocytes were the main source of *Cxcr4* within the LV tissue. The gene expression of the heterodimeric receptor *Cd74/Cd44*, depicted in blue and grey, showed that cardiomyocytes, cardiac fibroblasts and leukocytes express both receptor components. While leukocytes are the main source of *Cd74* expression, *Cd44* is well expressed in leukocytes and additionally in cardiac fibroblasts, both highly abundant in the infarct zone after MI. * significantly different compared with sham operated mice (**B**) or cardiomyocytes (**D**). * *p* < 0.05; ** *p* < 0.01; **** *p* < 0.0001.

The cell surface receptors CXCR4 and the heterodimer CD74/CD44 bind MIF and MIF-2. As illustrated in Figure 2A, CD74 and CD44 form a heterodimer in which CD74 is the virtual binding partner and CD44 the signaling component. First, we determined the basal expression levels of all three genes in healthy cardiac tissue. As shown in Figure 2C, *Cxcr4* revealed very low gene expression (0.0027 ± 0.0002-fold to *Cdkn1b*), whereas *Cd74* (1.0 ± 0.1-fold to *Cdkn1b*) and *Cd44* (0.19 ± 0.01-fold to *Cdkn1b*) are well expressed in cardiac tissue. Next, we analyzed whether their gene expression is upregulated after MI in the infarct zone 1 day and 5 days after LAD ligation. To analyze their relative regulation, we plotted the results as relative mRNA expression compared with sham operated animals in Figure 2B. The gene expression levels of all three cell surface receptors were highly increased in the infarct zone 1 day, as well as 5 days after MI.

To examine the cause of increased receptor expression within the IZ, we analyzed the basal gene expression of *Cxcr4*, *Cd74* and *Cd44* in different cell types. In Figure 2D, we plotted the ratio of the gene expression levels within the distinct cell type and the healthy LV tissue. Therefore, the gene expression of each receptor was normalized to its basal expression level in healthy cardiac tissue, shown in Figure 2C. Cardiomyocytes and cardiac fibroblasts did not express the MIF receptor *Cxcr4* but did express both receptor components for the heterodimeric MIF receptor *Cd74*/*Cd44*. As the ratio of *Cxcr4* expression in leukocytes is higher than one, those cells are the main contributor to the *Cxcr4* expression in LV tissue. Therefore, the infarct zone, representing inflamed cardiac tissue, showed highly increased *Cxcr4* expression in the acute (1 day) and subacute (5 days) phase after MI. Furthermore, *Cd74* as well as *Cd44* were expressed more highly in leukocytes than in healthy LV tissue. Therefore, the increased expression of *Cd74* and *Cd44* within the infarct zone is a result of recruited leukocytes.

### 3.3. Macrophage Migration Inhibitory Factor is Expressed in Murine Left Ventricular Tissue and Upregulated in Activated Cardiac Cells

**Figure 3:**
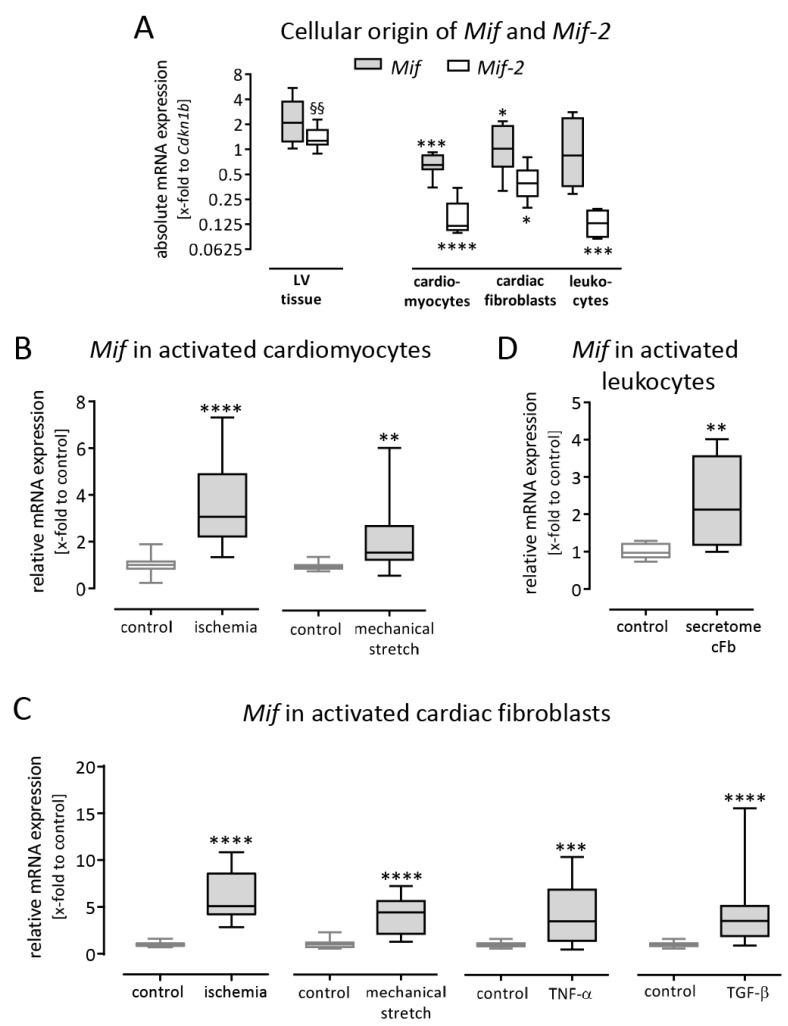
*Mif* is expressed in cardiac cells and upregulated after activation. (**A**) Basal gene expression of *Mif* and *Mif-2* was determined in LV tissue and individual cell cultures. Cardiac fibroblasts and leukocytes exhibited similar *Mif* expression compared with LV tissue, whereas cardiomyocytes expressed significantly less *Mif*. While *Mif* and *Mif-2* revealed similar expression levels in the LV tissue, considerably less *Mif-2* was determined in the investigated cardiac cell types representing not the main source of cardiac *Mif-2*. (**B**) Cultured cardiomyocytes were activated by simulated ischemia for 24 h (D-glucose was replaced by L-glucose and oxygen was reduced to 1%) or mechanically stretched with 10% elongation at a frequency of 1 Hz for 6 h. Ischemia as well as mechanical stretch induced *Mif* gene expression in cardiomyocytes. (**C**) Cardiac fibroblasts were exposed to simulated ischemia for 24 h or to mechanical stretch (10% elongation, 1 Hz) for 6 h. Both external stimuli resulted in increased *Mif* expression. Moreover, cardiac fibroblasts were stimulated with 10 ng/mL TNF-α or 5 ng/mL TGF-β for 6 h which led to increased gene expression levels of *Mif*. (**D**) Leukocytes were activated with cell culture supernatant derived from cardiac fibroblasts exposed to simulated ischemia. This conditioned medium induced the gene expression of *Mif* in leukocytes after 24 h. Basal gene expression levels are shown as absolute mRNA expression (x-fold to the housekeeping gene *Cdkn1b*) using the formula 2^−ΔCt^ and presented in box plots (min to max). Alteration of gene expression is depicted as relative mRNA expression (x-fold to control cells) using the formula 2^−ΔΔCt^. Gene expression data represent 2–8 independent experiments, each performed in 4–8 replicates. * significantly different compared with LV tissue or untreated cells as control; * *p* < 0.05; ** *p* < 0.01; *** *p* < 0.001; **** *p* < 0.0001. § significantly different compared with *Mif* expression in LV tissue; §§ *p* < 0.01.

While *Mif* and *Mif-2* had minor differences in expression levels in the LV tissue, distinct differences occurred identifying their origin. To determine the cellular source within the cardiac tissue, basal gene expression of *Mif* and *Mif-2* was measured in the LV tissue from healthy mice as well as in different cardiac cell types including cardiomyocytes, cardiac fibroblasts and leukocytes. To compare the gene expression, data are plotted as absolute mRNA expression x-fold to the housekeeping gene *Cdkn1b* and compared with the expression determined in LV tissue. As shown in Figure 3A, similar *Mif* expression levels were determined in cardiac fibroblasts (1.2 ± 0.2-fold to *Cdkn1b*) and leukocytes (1.2 ± 0.6-fold to *Cdkn1b*) compared with LV tissue (2.5 ± 0.3-fold to *Cdkn1b*), whereas significantly lower *Mif* expression was found in cardiomyocytes (0.7 ± 0.2-fold to *Cdkn1b*). In contrast, *Mif-2* expression in cardiomyocytes (0.16 ± 0.03-fold to *Cdkn1b*), cardiac fibroblasts (0.41 ± 0.08-fold to *Cdkn1b*) and leukocytes (0.13 ± 0.02-fold to *Cdkn1b*) was significantly lower compared with LV tissue (1.4 ± 0.1-fold to *Cdkn1b*). Accordingly, the investigated cardiac cell types did not represent the main source of cardiac *Mif-2* expression. Since *Mif* but not *Mif-2* was upregulated during acute MI, we further investigated different external stimuli to activate cardiomyocytes, cardiac fibroblasts and leukocytes detected as one cellular source of *Mif* within the LV tissue.

To further characterize *Mif* gene expression and its cellular origin, different stimuli were applied to cardiomyocytes, cardiac fibroblasts and leukocytes. Therefore, gene expression levels of *Mif* were assessed in cultured cardiomyocytes and cardiac fibroblasts exposed to simulated ischemia. As shown in Figure 3B,C, simulated ischemia induced *Mif* gene expression after 24 h in cardiomyocytes (3.6 ± 0.4-fold) as well as in cardiac fibroblasts (6.0 ± 0.5-fold). These findings are in line with the increased gene expression levels of *Mif* detected after myocardial infarction in mice (Figure 1A).

Next, we investigated whether mechanical stretch mimicking dilation of the cardiac ventricle post-MI influences *Mif* gene expression. Therefore, we applied cyclic mechanical stretch with an elongation of 10% and a frequency of 1 Hz to cultured cardiomyocytes and cardiac fibroblasts. As shown in Figure 3B,C, mechanical stretch significantly induced *Mif* gene expression after 6 h in cardiomyocytes (2.2 ± 0.5-fold) and cardiac fibroblasts (4.2 ± 0.7-fold) compared with the respective untreated control cells.

Moreover, during MI, cardiac fibroblasts are exposed to different pro-inflammatory and pro-fibrotic stimuli. Thus, we used TNF-α as a pro-inflammatory cytokine and TGF-β as a pro-fibrotic cytokine to stimulate cardiac fibroblasts. Subsequent gene expression analysis of stimulated cardiac fibroblasts revealed an effect of the recombinant TNF-α as well as TGF-β on the expression of *Mif*. As shown in Figure 3C, the pro-inflammatory cytokine TNF-α (4.2 ± 1.0-fold) as well as the pro-fibrotic growth factor TGF-β (4.8 ± 1.2-fold) led to elevated *Mif* expression.

Since we identified leukocytes as another source of MIF, we next aimed to investigate *Mif* expression in activated leukocytes. Therefore, cell culture supernatant derived from cardiac fibroblasts exposed to simulated ischemia was collected. Subsequently, isolated splenocytes representing invading leukocytes were activated using this conditioned medium followed by gene expression analysis. As visualized in Figure 3D, activated leukocytes revealed significantly higher gene expression levels of *Mif* (2.3 ± 0.4-fold) compared with untreated control cells.

### 3.4. Recombinant Macrophage Migration Inhibitory Factor Induced Paracrine Effects on Cardiac Fibroblasts but Not on Cardiomyocytes

**Figure 4:**
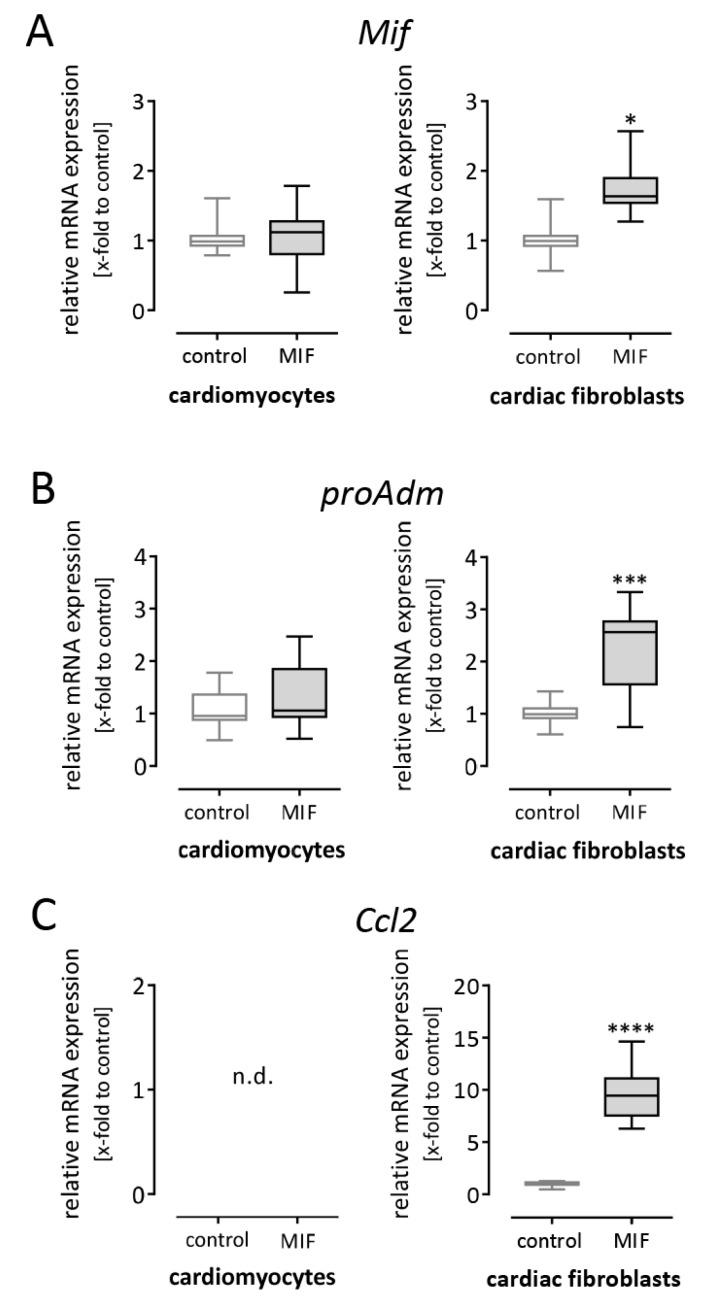
Recombinant MIF stimulates pro-inflammatory response in cardiac fibroblasts but not in cardiomyocytes. Cardiomyocytes and cardiac fibroblasts were stimulated with 50 ng/mL recombinant MIF for 24 h followed by gene expression analysis. (**A**) After MIF stimulation, cardiomyocytes did not alter the gene expression of *Mif*, whereas in cardiac fibroblasts MIF stimulation in turn increased the gene expression of *Mif* and may amplify its effects. (**B**) The expression of *proAdm* was not significantly increased in cardiomyocytes but in cardiac fibroblasts. (**C**) The pro-inflammatory chemokine *Ccl2* was not detectable in cardiomyocytes. In contrast, cardiac fibroblasts respond to MIF stimulation with highly increased *Ccl2* expression. Gene expression levels are shown as relative mRNA expression (x-fold to control cells) using the formula 2^−ΔΔCt^ and presented in box plots (min to max). Gene expression data represent three independent experiments, each performed in 6–12 replicates. * significantly different compared with untreated cells as control; * *p* < 0.05; *** *p* < 0.001; **** *p* < 0.0001; n.d. not detectable.

As shown in Figure 1 and Figure 3, *Mif* expression is induced by MI in cardiac tissue and by various external stimuli in different cardiac cell types. Next, we aimed to identify possible paracrine or autocrine effects of MIF protein regarding cardiac inflammation. Therefore, cardiomyocytes as well as cardiac fibroblasts were stimulated with 50 ng/mL recombinant MIF protein for 24 h followed by gene expression analysis. First, we examined whether MIF stimulation can in turn induce *Mif* gene expression in cardiomyocytes and cardiac fibroblasts. As shown in Figure 4A, cardiac fibroblasts but not cardiomyocytes increased *Mif* expression after MIF stimulation and therefore may amplify the effects of MIF secreted by different cardiac cell types.

Further, we investigated the paracrine or autocrine effects of MIF on cardiomyocytes and cardiac fibroblasts regarding inflammation. After MIF stimulation, gene expression of pro-inflammatory *proAdm* and *Ccl2* was analyzed. As shown in Figure 4B,C, gene expression levels of *proAdm (*2.3 ± 0.2-fold to *Cdkn1b*) and *Ccl2 (*9.6 ± 0.5-fold to *Cdkn1b*) were significantly elevated in cardiac fibroblasts after MIF stimulation. In contrast, in cardiomyocytes, increased *proAdm* expression was not determined and the expression of the chemokine *Ccl2* was not detectable. Altogether, cardiomyocytes did not respond to MIF stimulation, whereas cardiac fibroblasts amplified *Mif* expression and increased their expression of pro-inflammatory cytokines.

### 3.5. Gene Expression in Cardiac Fibroblasts is Altered after Stimulation with Macrophage Migration Inhibitory Factor and Simulated Ischemia

As shown in Figure 3, simulated ischemia induced *Mif* expression in cardiomyocytes, as well as in cardiac fibroblasts. In contrast, MIF stimulation in turn induced pro-inflammatory gene expression in cardiac fibroblasts but not in cardiomyocytes (Figure 4). Therefore, we further examined cardiac fibroblasts regarding the effects of ischemia in the presence or absence of recombinant MIF protein to uncover possible paracrine effects of MIF during MI.

**Figure 5:**
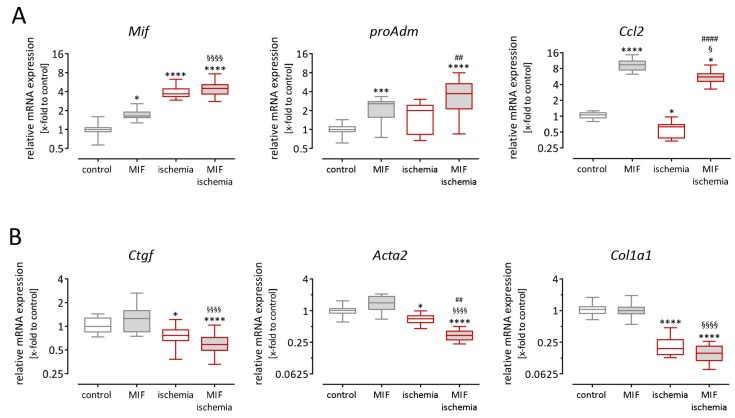
Pro-inflammatory and fibrotic signaling in cardiac fibroblasts exposed to ischemia in the presence of MIF. Murine cardiac fibroblasts were stimulated using 50 ng/mL recombinant murine MIF or simulated ischemia for 24 h. (**A**) Pro-inflammatory gene expression of *Mif*, *proAdm* and *Ccl2* was quantified. The expression of *Mif* and *proAdm* was increased by stimulation using recombinant MIF and simulated ischemia. The combination of both stimuli revealed additive effects and the gene expression was further increased. The expression of *Ccl2* was increased by stimulation using recombinant MIF but decreased by ischemia. Combining both stimuli, the MIF-induced *Ccl2* expression was attenuated by applying ischemia. (**B**) MIF stimulation did not alter the expression of the fibrotic genes *Ctgf*, *Acta2* and *Col1a1*, whereas the gene expression was significantly reduced during simulated ischemia. The combination of both stimuli resulted in further reduced expression levels. Data from cardiac fibroblasts under simulated ischemic conditions are plotted with red borders. Additional treatment is indicated using grey-filled bars. Gene expression levels are shown as relative mRNA expression (x-fold to control cells) using the formula 2^−ΔΔCt^ and presented in box plots (min to max). Gene expression data represent 3 independent experiments, each performed in 6–12 replicates. * significantly different compared with untreated cells as control; * *p* < 0.05; *** *p* < 0.001; **** *p* < 0.0001. § significantly different compared with MIF; § *p* < 0.05; §§§§ *p* < 0.0001. # significantly different compared with ischemia; ## *p* < 0.01; #### *p* < 0.0001.

Cardiac fibroblasts were exposed to 50 ng/mL recombinant MIF alone and to simulated ischemia in the presence or absence of recombinant MIF for 24 h. Subsequently, gene expression levels of inflammatory and fibrotic genes were analyzed. As shown in Figure 5A, recombinant MIF resulted in increased gene expression levels of the pro-inflammatory mediators *Mif*, *proAdm* and *Ccl2*. While *Mif* and *proAdm* expression was approximately 2-fold greater, the enhancement of *Ccl2* expression reached 10-fold compared with untreated cells. Next, we examined the influence of ischemia alone. As shown in Figure 5A, *Mif* expression was 4-fold greater and therefore even higher compared with stimulation using recombinant MIF. Furthermore, the expression of *proAdm* was slightly but not significantly increased during ischemia. In contrast, ischemia resulted in significantly reduced expression of *Ccl2* revealing the opposite impact of MIF protein stimulation. Simulated ischemia in the presence of recombinant MIF protein triggered additive effects in cardiac fibroblasts. While the expression of *Mif* and *proAdm* was further increased, the expression of *Ccl2* was attenuated from 9.6-fold increased expression after MIF stimulation to 5.6-fold increased expression after ischemia in the presence of MIF.

As shown in Figure 5B, we further examined the expression of pro-fibrotic genes in cardiac fibroblasts, namely *Ctgf*, *Acta2* and *Col1a1*. Using recombinant MIF to stimulate cardiac fibroblasts, no altered pro-fibrotic gene expression was found. In contrast, simulated ischemia reduced the expression of *Ctgf*, *Acta2* and *Col1a1* in cardiac fibroblasts. While MIF stimulation itself had no influence on pro-fibrotic gene expression, simulated ischemia in the presence of recombinant MIF revealed a further reduction of pro-fibrotic gene expression compared with ischemia in the absence of MIF.

## 4. Discussion

Since MIF is known as a pleiotropic cytokine, it is involved in several inflammatory diseases such as MI. In mice, gene and protein expression of MIF increased during the acute phase of MI. This was associated with increased expression of other pro-inflammatory cytokines such as *Tnf-α* and *Ccl2*, representing the inflammatory phase. Our in vitro experiments revealed that cardiomyocytes as well as cardiac fibroblasts increased *Mif* expression during simulated ischemia and other external stimuli. Utilizing recombinant MIF protein, pro-inflammatory effects were identified in cardiac fibroblasts but not in cardiomyocytes. Since MIF in turn induced gene expression of *Mif* in cardiac fibroblasts, they may amplify the cardio-protective function of MIF on cardiomyocytes.

### 4.1. Cardio-Protective Function of Macrophage Migration Inhibitory Factor

The function of MIF during MI is complex, as well as time dependent [38]. Especially during acute ischemia, MIF mediates cardio-protection mainly through the CD74/CD44 receptor complex utilizing several mechanisms: AMPK activation leading to increased glucose uptake [31], inhibiting JNK to suppress apoptotic signaling [39] and reducing oxidative stress [40]. In line with these findings, using an ischemia/reperfusion model (15 min ischemia/4 h reperfusion), MIF deficient mice revealed increased infarct sizes [31]. Despite its cardio-protective functions, in a prolonged ischemia/reperfusion model (60 min ischemia/24 h reperfusion), MIF deficiency reduced infarct sizes and cardiomyocyte apoptosis since the inflammatory response was suppressed in MIF KO mice. In other cardiac diseased models, such as pressure overload-induced cardiac hypertrophy [41], diabetic cardiomyopathy induced by type 1 diabetes [42] and doxorubicin-induced cardiomyopathy [43], MIF deficiency exacerbated the impaired cardiac function and lead to increased mortality. These studies emphasize the cardio-protective function of MIF in the absence of aggravating cardiac inflammation. Besides its structural similarities to MIF, MIF-2 binds to the CD74/CD44 receptor complex and recapitulates all important actions of MIF [8,26,27]. In our MI mouse model opposing expression levels were determined after MI. Therefore, we suggest no crucial cardio-protective role of MIF-2 during acute MI.

### 4.2. Cellular Origin of Macrophage Migration Inhibitory Factor after Myocardial Infarction

Myocardial infarction patients are known to have elevated plasma levels of MIF, which are predictive for infarct size [30], but the molecular actions of MIF and its role during MI remain unclear. In our experiments, we first determined the basal expression of *Mif* in cardiac cells including cardiomyocytes, cardiac fibroblasts and splenocytes representing a variety of leukocytes. Compared with the *Mif* expression in healthy LV tissue, similar expression levels were found in cardiac fibroblasts as well as in leukocytes, whereas cardiomyocytes exhibited minor *Mif* expression. The structurally related *Mif-2* was expressed to a slightly lower extent in healthy LV tissue compared to *Mif*. The cells investigated here exhibited considerably less *Mif-2* expression suggesting other additional sources. Less is known about the role of MIF-2 in diseased animal models, but regarding our data, we suggest an inferior impact compared to MIF.

Next, we exposed the investigated cell types to different external stimuli. During simulated ischemia *Mif* expression increased in cardiomyocytes and cardiac fibroblasts. Furthermore, to mimic MI, conditioned medium derived from cardiac fibroblasts exposed to ischemia was used to activate leukocytes resulting in increased *Mif* expression as well. It was already reported that long term chronic hypoxia induces *Mif* expression in rat cardiomyocytes [20]. Regarding our results, *Mif* expression during MI is not only caused by cardiomyocytes, but more prominently by cardiac fibroblasts. In addition to simulated ischemic conditions we applied both cardiac cell types, cardiomyocytes and cardiac fibroblasts, to mechanical stretch mimicking post-MI cardiac dilation. Interestingly, both cell types increased *Mif* expression as a result of mechanical stretch.

It is well known that during the acute phase after MI, inflammatory cells invade the injured myocardium. Therefore, we stimulated cardiac fibroblasts with TNF-α and TGF-β known to be secreted by activated immune cells. Both proteins led to increased *Mif* expression in cardiac fibroblasts. Since cardiac fibroblasts express *Mif* to a higher extent, increased *Mif* expression during MI might be caused by ischemia on the one hand and by subsequently developed cardiac inflammation on the other hand.

### 4.3. Macrophage Migration Inhibitory Factor and Cardiac Inflammation after Myocardial Infarction

In general, MIF is known as a pleiotropic cytokine and this might be applicable to MIF-2 as well [8,26]. Despite its cardio-protective functions, MIF deficiency becomes beneficial when cardiac inflammation contributes to myocardial injury. After prolonged and severe ischemia/reperfusion injury, reduced cytokine expression and less leukocyte infiltration was observed in *Mif*^−/−^ mice compared to wild-type (WT) [32]. Macrophage Migration Inhibitory Factor itself can elicit leukocyte recruitment through the CXCR2/4 receptors [23]. Macrophages derived from WT mice migrate towards homogenized infarct tissue from WT. This effect was significantly reduced in response to homogenized infarct tissue from *Mif*^−/−^ mice, demonstrating less chemokine expression in *Mif*^−/−^ mice after MI. Interestingly, when *Mif*^−/−^ macrophages were exposed to WT infarct tissue, migration was also lower than WT macrophages [44].

In contrast to increased *Mif* expression levels during the acute phase after MI, characterized by arising cardiac inflammation, *Mif-2* expression was significantly decreased. These data suggest complementary effects on inflammation, but so far, nothing is known about the function of MIF-2 during MI. In line with our observation, *Mif-2* expression was decreased in inflammatory adipose tissue compared with healthy controls [45], whereas *Mif* expression was increased and may mediate the recruitment of inflammatory cells [46]. In mice, macrophage recruitment was induced by lipopolysaccharide (LPS) injection and was further increased when MIF was injected additionally. In contrast, additional injection of MIF-2 does not lead to further increased macrophage recruitment compared with LPS injection alone. Regarding our data, we suggest that MIF-2 does not contribute to the development of cardiac inflammation.

**Figure 6:**
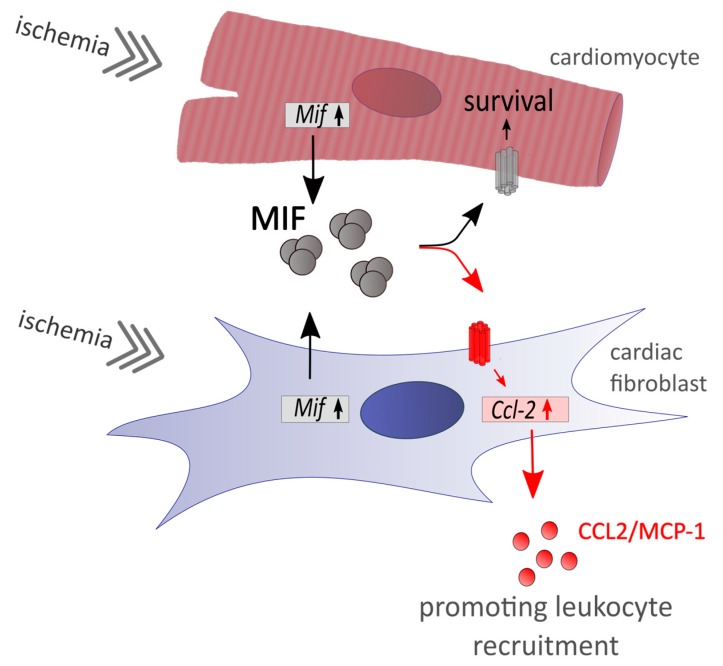
While ischemia induces *Mif* expression in both cardiac cell types, cardiomyocytes and cardiac fibroblasts, secreted MIF protein revealed different functions. Well characterized cardio-protective effects of MIF are only identified in cardiomyocytes, whereas cardiac fibroblasts may serve as sentinel cells to promote the pro-inflammatory effect of MIF by expressing chemoattractive chemokines.

Besides its own chemoattractant properties, recombinant MIF induced chemokine expression in cardiac fibroblasts rather than provoking expression of matrix proteins required for scar stabilization after MI. On cardiac fibroblasts, MIF led to increased chemokine expression promoting leukocyte recruitment on the one hand, but in turn induced *Mif* expression itself serving as cardio-protection on cardiomyocytes on the other hand (Figure 6). Since circulating MIF directly facilitates leukocyte recruitment towards inflammation, MIF represents a suitable target to avoid major adverse effects. Using a MIF-neutralizing antibody, macrophage and T-cell content were reduced in atherosclerotic plaques [23].

### 4.4. Macrophage Migration Inhibitory Factor—Utilizing Its Cardio-Protective Function but Treating the Pro-Inflammatory Function?

Macrophage Migration Inhibitory Factor shows cardio-protective functions but provokes cardiac inflammation which in turn is known to be detrimental for the myocardial tissue after MI. Since MIF deficiency becomes beneficial regarding cardiac inflammation, anti-MIF interventions might be a potential therapeutic option following MI [5]. Anti-MIF intervention using a MIF antagonist significantly reduced the incidence of cardiac rupture that occurred at 3–4 days after MI [5]. Other inflammatory diseases also benefit from administration of anti-MIF antibodies. In mice subjected to peritonitis sepsis, administration of anti-MIF antibodies could significantly increase the survival rate [47].

Since inflammatory response is essential for the subsequent healing processes, identifying the extent and time frame of anti-inflammatory treatments is still a great challenge [5].

## 5. Conclusions

The presented data show that MIF was expressed by resident cardiac cells during MI. In vitro, *Mif* expression was induced by different external stimuli in cardiomyocytes and cardiac fibroblasts. Addition of recombinant MIF protein increased the expression of pro-inflammatory genes in cardiac fibroblasts including *Mif* expression itself. Thereby, cardiac fibroblasts may amplify *Mif* expression during ischemia supporting cardiomyocyte survival, but increased chemokine expression may promote leukocyte recruitment leading to aggravated cardiac inflammation.

## Figures and Tables

**Table 1:** Antibodies for immunofluorescence detection.

Antibody	Species	Dilution	Company	Catalogue No.#
**Primary antibodies**				
anti-MIF (FL-115)	Rabbit	1:100	Santa Cruz	sc-20121
anti-MCP-1 (ECE.2)	Rat	1:50	abcam	ab8101
**Secondary antibodies**				
anti-rabbit IgG (H + L) Alexa-568	Donkey	1:500	Life technologies	A10042
anti-rat IgG (H + L) Alexa-488	Donkey	1:500	Life technologies	A21208

**Table 2:** Gene expression assays purchased from Life technologies.

Gene Symbol	Gene Name	Assay ID
m*Acta2*	α smooth muscle actin	Mm00725412_s1
m*Ccl2*	chemokine (C-C motif) ligand 2	Mm99999056_m1
m*Cd44*	cluster of differentiation 44	Mm01277161_m1
m*Cd74*	cluster of differentiation 74	Mm00658576_m1
m*Cdkn1b*	cyclin-dependent kinase inhibitor 1B	Mm00438167_g1
m*Col1a1*	collagen type I, α 1	Mm01302043_g1
m*Ctgf*	connective tissue growth factor	Mm00515790_g1
m*Cxcr4*	chemokine (C-X-C motif) receptor 4	Mm01996749_s1
m*Ddt (Mif-2)*	D-dopachrome tautomerase	Mm00515641_m1
m*Mif*	macrophage migration inhibitory factor	Mm01611157_gH
m*proAdm*	pro-adrenomedullin	Mm00437438_g1
m*Tnf*α	tumor necrosis factor α	Mm00443258_m1
